# Anesthetic Management of Pheochromocytoma Resection in Adults with Single Ventricle Physiology

**DOI:** 10.7759/cureus.1928

**Published:** 2017-12-08

**Authors:** Giancarlo Suffredini, Natalia Diaz-Rodriguez, Krishnan Chakravarthy, Aarti Mathur, Heather K Hayanga, Steve M Frank, Richard E Ringel, Stephen Freiberg, Viachaslau M Barodka, Jochen Steppan

**Affiliations:** 1 Anesthesiology and Critical Care Medicine, The Johns Hopkins University School of Medicine; 2 Anesthesia and Critical Care Medicine, The Johns Hopkins University School of Medicine; 3 Department of Anesthesia, Critical Care and Pain Medicine, University of California, San Diego; 4 Surgery, The Johns Hopkins University School of Medicine; 5 Department of Anesthesiology, West Virginia University School of Medicine; 6 Pediatrics, The Johns Hopkins University School of Medicine

**Keywords:** single ventricle, fontan, pheochromocytoma, neuraxial

## Abstract

Survival rates for patients with palliated congenital heart disease are increasing, and an increasing number of adults with cyanotic congenital heart disease (CCHD) might require surgical resection of pheochromocytoma-paraganglioma (PHEO-PGL). A recent study supports the idea that patients with a history of CCHD and current or historical cyanosis might be at increased risk for developing PHEO-PGL. We review the anesthetic management of two adults with single-ventricle physiology following Fontan palliation presenting for PHEO-PGL resection and review prior published case reports. We found the use of epidural analgesia to be safe and effective in the operative and postoperative management of our patients.

## Introduction

The Fontan procedure is the most common staged palliation performed for patients with any type of single ventricle. Because improvements in surgical, perioperative, and cardiac management have resulted in increased survival, these patients are presenting more frequently for non-cardiac surgery. Further, these patients have a higher incidence of pheochromocytoma-paragangliomas (PHEO-PGL) in adulthood [1]. These catecholamine-secreting neuroendocrine tumors can be life-threatening, especially in patients with single-ventricle physiology due to increases in systemic and pulmonary vascular resistance that can lead to cardiovascular collapse. We review our anesthetic management of two adults with single-ventricle physiology who had prior Fontan palliation and required PHEO-PGL resection. The patients have provided written consent to publish this case report.

## Case presentation

**Figure 1:**
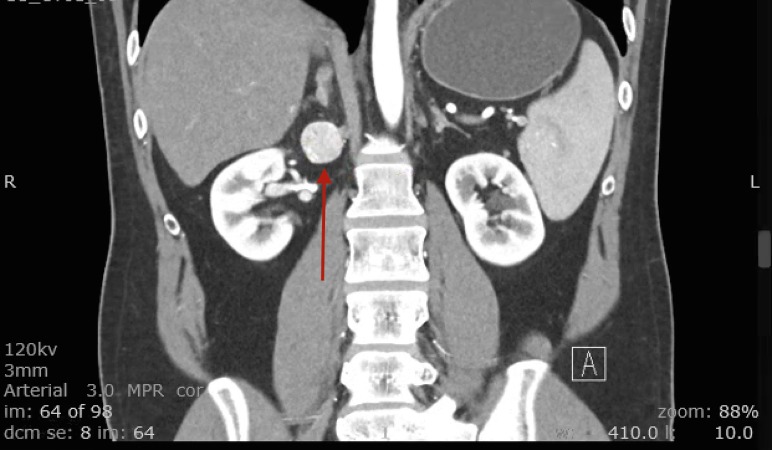
CT Scan Patient One Red arrow shows hyper-intense lesion medial to the right kidney, suggestive of a paraganglioma

**Figure 2:**
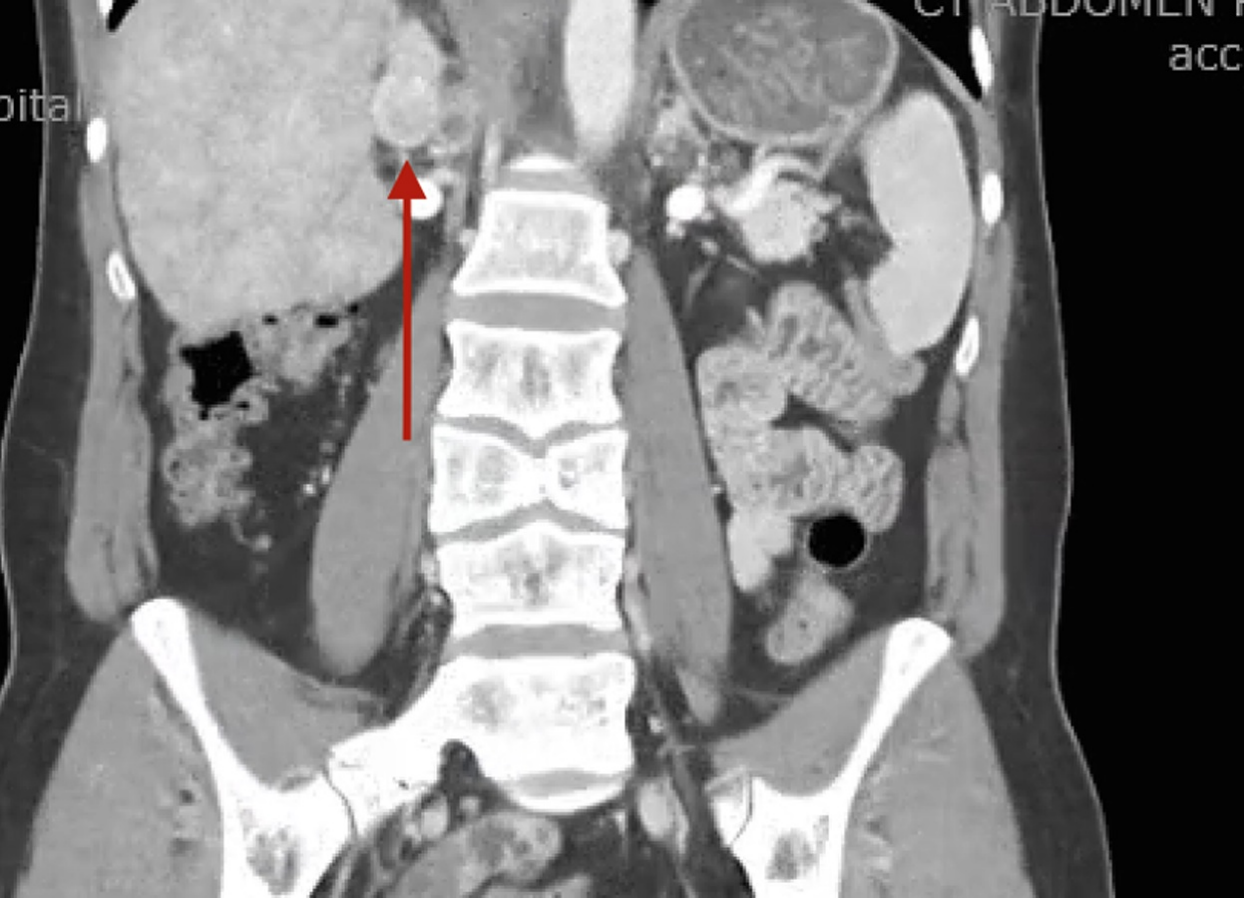
CT Scan Patient Two Red arrow shows large hypervascular right-sided adrenal mass

Patient one is a man with tricuspid atresia had undergone a classic Fontan palliation as a child and subsequently underwent a Fontan revision to an extracardiac tunnel (extracardiac inferior vena cava to right pulmonary artery conduit). At 36 years, while undergoing a computerized tomography (CT) scan to screen for cardiac cirrhosis, a hyper-intense lesion medial to the right kidney suggestive of a paraganglioma was discovered (Figure 1). Patient two is a 35-year-old woman who was a Jehovah’s Witness had a double inlet left ventricle with straddling tricuspid valve (hypoplastic right ventricle), restrictive ventricular septal defect (VSD), pulmonary stenosis, and bilateral superior vena cava (SVC). She underwent an extracardiac lateral Fontan and bilateral bidirectional Glenn shunt as a child. At age 35, she presented to the emergency department complaining of right-sided flank pain, diaphoresis, and palpitations. A CT scan of the abdomen revealed a large hypervascular right-sided adrenal mass (Figure 2) and a small enhancing lesion in the left lobe of the liver.

Biochemical testing and radionuclide scan were consistent with either a paraganglioma or pheochromocytoma in both patients, and they were started on doxazosin. They were consequently evaluated by our high-risk cardiac disease team, which is led by a cardiac anesthesiologist and includes specialists from adult and pediatric cardiac anesthesiology, cardiology, electrophysiology, surgery, and adult congenital heart disease (CHD) specialists. Perioperative management in both patients was similar. The patients were optimized medically and brought to the operating room for tumor resection. After mild sedation, a thoracic epidural was placed at the T8-9 level, standard monitors and a radial arterial catheter were placed prior to induction. Anesthesia was induced with fentanyl, ketamine, and midazolam, and supplemented with 0.5% isoflurane. The patients were intubated after receiving vecuronium and ventilated with low tidal volumes (6 ml/kg) to minimize mean airway pressures and a fraction of inspired oxygen (FiO2) of 60%, a positive end-expiratory pressure (PEEP) of 5 cm H2O, and a rate adjusted to maintain the end-tidal carbon dioxide (ETCO2) at 35 mmHg. A transesophageal echocardiographic (TEE) probe was introduced, and central venous access was obtained with a double-lumen catheter via the right internal jugular vein. TEE guidance was used to insert the catheter to a depth of 10 cm, and care was taken not to disturb the Glenn anastomosis. An 18-gauge peripheral IV was inserted in the right forearm. Anesthesia was maintained with 0.5-0.8% isoflurane and an epidural infusion of 0.125% bupivicaine. For the patient who was a Jehovah’s Witness, an autologous blood recovery system was connected in a continuous manner with the patient for blood salvage [2].

**Figure 3:**
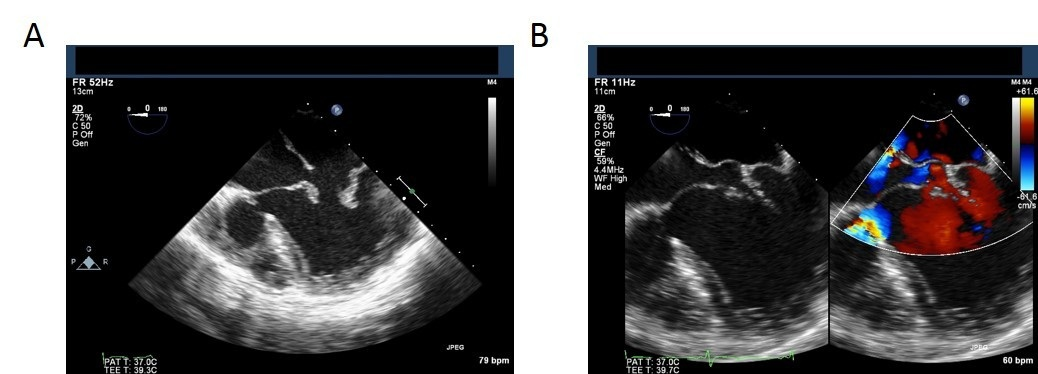
Intra-operative Transesophageal Echocardiogram Patient 2 (A) Double inlet left ventricle (B) ASD, VSD and double outlet left ventricle

The central venous pressure (CVP), closely reflecting pulmonary artery pressure in Fontan physiology, was monitored and maintained at each patient’s baseline. TEE imaging was used throughout the case to monitor volume status and cardiac function. A representative TEE image of patient two is shown in Figure 3. Both surgical procedures proceeded uneventfully with only brief periods of hypertension, requiring treatments with a vasodilator (sodium nitroprusside). After tumor removal, the patients remained normotensive without vasopressors or inotropes. The patient received 800 mL and 2 liters of crystalloid solution respectively, with minimal blood loss. No blood products, including the autologously recovered blood, were transfused. Neuromuscular blockade was reversed using glycopyrrolate and neostigmine, and the patients were extubated in the operating room and transported to an intensive care unit. Thoracic epidurals were removed on postoperative day 3 and provided analgesia with limited use of opioids during the immediate postoperative period. Both patients were discharged home on postoperative day five.

## Discussion

Current evidence suggests that an increasing number of patients with CHD survive into adulthood. A subset of those patients, those with cyanotic CHD (CCHD) might have an increased incidence of PHEO-PGL. A recent large multicenter series and population-based observational study of patients with CCHD supports the relationship between exposure to chronic hypoxemia (defined as at least one year with arterial saturation ≤ 92%) in patients with CCHD and development of PHEO-PGL [1]. However, the timing and duration of hypoxemia as a risk factor in the development of PHEO-PGL in such patients is unknown. In general, patients with single-ventricle physiology who are palliated with a Fontan operation are hypoxic for a few years as a child, between the Glenn placement and the Fontan completion (some are also hypoxic at birth for a short period of time). However, after completion of the Fontan, patients should have near normal oxygen saturations unless they develop significant aorto-pulmonary collaterals or they have a large fenestration and pulmonary hypertension.

Patients with single-ventricle physiology are preload-dependent and require low pulmonary and left atrial pressures to facilitate blood flow across the lungs and to maintain cardiac output. Therefore, goals of perioperative anesthetic management include maintaining normovolemia, baseline CVP, sinus rhythm, and low pulmonary artery pressures; supporting contractility of the systemic ventricle; avoiding air in intravenous lines; and using postoperative pain control that will not compromise ventilation.

We elected to use TEE in both patients to monitor placement of the central line, to help guide volume management, and assess potential ventricular dysfunction from catecholamine release during the operation. A major difference between our anesthetic management and that described in prior reports was the inclusion of epidural analgesia as part of our intraoperative anesthetic, which helped to facilitate early extubation, and for postoperative analgesia, which helped to avoid splinting and the respiratory depressant effects of opioid-based analgesia. Avoiding postoperative hypoventilation is of particular importance in this patient population because complications such as atelectasis and pneumonia can lead to hypoxia and hypercarbia which can increase pulmonary pressures, decrease the transpulmonary gradient, and ultimately compromise cardiac output.

Safe use of epidural analgesia has not been described in patients with Fontan physiology undergoing resection of pheochromocytoma. Concerns regarding the use of epidural analgesia in these patients include the unpredictable hemodynamic effects of vasodilation and bradycardia, epidural collaterals leading to intravenous catheter migration, and the potential for epidural hematoma as a result of elevated venous pressures and coagulation abnormalities. To compare our approach with prior publications, a systematic literature search of PubMed and Embase was performed using the following search terms: Search: ((("single ventricle") OR “Fontan”)) AND ("Pheochromocytoma"[Mesh] OR "Paraganglioma"[Mesh] OR "Pheochromocytoma" OR "Paraganglioma" OR "Pheochromocytomas" OR "Paragangliomas"). We included only articles published before December 2016 and excluded non-English articles (Table 1).

**Table 1:** Review of Case Reports Describing Anesthetic Management of Patients with Single-Ventricle Physiology for Resection of Pheochromocytoma ASD, atrial septal defect; AV, atrioventricular; CVP, central venous pressure; EV, ejection fraction; ICU, intensive care unit; LOS, length of stay; PACU, post-anesthesia care unit; PCC, pheochromocytoma; POD, postoperative day; OR, operating room; RA, right atrium; RV, right ventricle; SVC, superior vena cava; TEE, transesophageal echocardiographic; VSD, ventricular septal defect.

Case	Tjeuw, et al. [3]	Sparks, et al. [4]	Yuki, et al. [5]	Latendresse, et al. [6]	Cherqaoui, et al. [7]	Lee, et al. [8]	Haile, et al. [9]
Age, years (gender)	11 (F)	27 (F)	24 (M)	11 (M)	13 (M)	18 (M)	25 (M)
Underlying disease and palliation	Dextro-transposition of the great arteries, tricuspid atresia, ASD, pulmonary artery stenosis. Occluded Waterston shunt. Stenotic left Blalock–Taussig shunt. Patent right Blalock–Taussig shunt.	Single right ventricle with discontinuous pulmonary arteries. Superior cavopulmonary anastomosis to right upper and middle pulmonary arteries. Central aorto-pulmonary graft to right lower and left pulmonary arteries.	Holmes type doublet inlet single left ventricle, normally related pulmonary arteries without pulmonary stenosis. Right atrial appendage to main pulmonary artery modified Fontan.	Large VSD resulting in single-ventricle physiology.	Tricuspid atresia, pulmonary artery stenosis, dysplastic single AV valve, and duplication of SVC. Blalock–Taussig shunt. Modified Glenn shunt.	Single right ventricle, complete endocardial cushion defect, corrected transposition of great arteries, right isomerism, supracardiac type total anomalous pulmonary venous return. Bidirectional cavo-pulmonary shunt followed by Fontan.	Pulmonary atresia status post Fontan procedure
Echo	None described	Mild regurgitation at tricuspid, mitral, and aortic valves and mildly depressed RV systolic function	Severe RA dilation. “Good” LV function. No aortic or mitral valve regurgitation	Situs inversus Dextrocardia “Good” ventricular function No visible Rà L communications	“Good” ventricular systolic function and mild AV valve regurge Cavopulmonary connections patent and continuous pulmonary blood flow	EF 50%, mild AV valve regurge Normal wall motion No obvious stenosis within the Fontan pathway or pulmonary vessels	EF 53%, mild AV valve regurgitation
Induction	Fentanyl (50 mcg/kg), diazepam (0.5 mg/kg), vecuronium (0.15 mg/kg)	Etomidate, sufentanyl, rocuronium	Etomidate, fentanyl, vecuronium	Etomidate (70 mcg/kg), rocuronium (1.5 mcg/kg), remifentanil (2-3 mcg/kg)	Premedication with midazolam, (0.3 mg/kg). Hydroxyzine (2 mg/kg), propofol (2 mg/kg), remifentanil (1 mcg/kg), atracurium (0.5 mg/kg)	Midazolam (2.5 mg), remifentanil (40 mcg), etomidate (10 mg), rocuronium (50 mg)	Fentanyl, lidocaine, propofol, vecuronium
Maintenance	Fentanyl boluses and vecuronium	Sufentanyl infusion, low concentration of isoflurane	Fentanyl, midazolam, pancuronium, and a low concentration of isoflurane	Sevoflurane (1.8%), remifentanil (0.1-0.3 mcg/kg/ min)	Remifentanil, sevoflurane	Sevoflurane, remifentanil	Remifentanyl, isoflurane, continuous nebulized epoprostenol, and milrinone
Central access	Yes, right internal jugular	No, femoral attempted but no success	Yes, location not specified	Yes, internal jugular and femoral	Yes, left subclavian under fluoroscopy	Yes, right internal jugular	Not specified
Baseline CVP (mmHg)	12	Not described	20	13-15	Not described	15	Not described
TEE	No	No	Yes	Yes	No	Yes	No
Arterial line	Right radial	Right radial	Yes, side not given	Radial, side not given	Right femoral	Right radial	Not described
Epidural	No	No	No	No	No	No	No
Laparoscopic or open (time of surgery)	Open (2.5 hours)	Open (3 hours)	Open (4 hours)	Open (8 hours)	Open (5 hours)	Laparopscopic; converted to open (8 hours)	Open
Complications	None	None	Phenylephrine infusion x 4 days. Multiple cardioversions and amiodarone for atrial arrhythmias	Volume overload atelectasis, pulmonary effusions, ascites, and paralytic ileus	None	None	Undiagnosed PCC at time of surgery
Extubation location	ICU on POD1	OR	ICU 1 week post-op	OR	ICU 2 hour post-op	ICU 12 hour post-op	OR
LOS	8 days ICU vs hospital length not differentiated	3 days ICU vs hospital stay not differentiated	19 days	7 days in ICU; 21 days on floor	24 h in ICU; 6 days on floor	3 days in ICU; 10 days floor	PACU recovery; LOS not specified

We elected to obtain central venous access primarily for vasoactive infusions. Placing a catheter in the veins of the neck carries a risk of catheter-associated thrombosis and associated obstruction of the Glenn anastomosis. We entered the right internal jugular vein from a more cephalad position and used TEE to ensure that the wire and the catheter were positioned above the Glenn anastomosis. It is important to note that the pressures obtained from central venous access essentially reflect pulmonary artery pressures in this patient population. An alternative method validated to measure pulmonary artery pressures in patients with unobstructed cavopulmonary or Fontan connections is by transducing pressure from an upper extremity [10]. Although this method avoids the potential complication of thrombosis at the Glenn anastomosis, accurate measurements rely on an unobstructed path from the peripheral vein to the pulmonary artery, and is limited in patients who have significant veno-venous collaterals [10].

Lastly, perioperative fluid management is important in these patients because an adequate intravascular volume is required to keep CVP high enough to facilitate blood flow through the pulmonary circulation. Decreased preload can result in decreased pulmonary blood flow and cardiac output. Given the lack of pulsatile pulmonary blood flow, the absence of a compliant reservoir for the systemic venous circulation (i.e., the right ventricle) and the series arrangement of all resistances and pressures in the Fontan circulation, an increased preload simultaneously results in an increased afterload increasing the workload for the systemic ventricle. Volume overload may lead to atrial or ventricular distension, predisposing patients to atrial fibrillation, and resulting in a decreased transpulmonary gradient. The goal, therefore, is to maintain euvolemia in these patients, using both TEE guidance and CVP monitoring to closely follow volume status.

## Conclusions

In summary, we describe the anesthetic management of two adult patients with a single-ventricle and Fontan physiology who underwent open resections of adrenal paraganglioma. Our anesthetic technique included epidural placement for intra- and postoperative pain control, TEE guidance for fluid management, and the goal of euvolemia and early extubation. This approach allowed us to discharge both patients after five days, which appears to be relatively early compared to descriptions in prior case reports.
